# Differentiation and Grouping Practices as a Response to Heterogeneity – Teachers’ Implementation of Inclusive Teaching Approaches in Regular, Inclusive and Special Classrooms

**DOI:** 10.3389/fpsyg.2021.676482

**Published:** 2021-09-10

**Authors:** Katharina-Theresa Lindner, Lena Nusser, Karin Gehrer, Susanne Schwab

**Affiliations:** ^1^Department of Education, University of Vienna, Vienna, Austria; ^2^Leibniz Institute for Educational Trajectories, Bamberg, Germany; ^3^Optentia Research Focus Area, North-West University, Vanderbijlpark, South Africa

**Keywords:** differentiation, grouping, students’ heterogeneity, teaching practices, inclusive education, special education

## Abstract

Addressing students’ individual needs is a crucial component of inclusive teaching. However, empirical evidence comparing practices such as differentiation and grouping strategies within inclusive, regular and special classes is still lacking. The present study contrasts these settings using data from the German National Educational Panel Study (NEPS). Data from 1034 teachers (755 regular, 89 inclusive, 190 special teachers) teaching the subject German in secondary school (grade 5 to grade 8) were used. Results show the highest use of differentiation in special school classes. Teachers’ use the majority of grouping practices to a similar extent when comparing the three educational settings. Class size and the number of students with migration background were predictors for teachers’ use of differentiation, whereas patterns of grouping strategies were predicted by students’ gender and teachers’ experience.

## Introduction

Inclusive teaching practices lead to an educational emphasis on the consideration of the diversity of students characteristics and the avoidance of producing learning barriers for students who have traditionally been disadvantaged, e.g., in consequence of individual characteristics such as a low socio-economic background, ethnic and linguistic minority background, special educational needs or gender (e.g., Ainscow and Messiou, 2018; Schwab, 2019; United Nations Educational Scientific and Cultural Organization, 2020). In this context, Molbaek (2018) distinguishes four dimensions of inclusive teaching practice: (1) frame, (2) relation, (3) organization, and (4) didactics. Hattie (2009) describes teachers’ primary focus regarding students’ inclusion on didactic and organizational features of teaching. The organizational dimension encompasses strategies and approaches considering inclusive teaching such as an elaborated usage of grouping practices (Vaughn et al., 2007). The didactic dimension deals with the actual implementation of inclusion in everyday school life including reactive didactic approaches as a response to the heterogeneity of students’ educational needs such as differentiated and individualized instruction (Tomlinson, 2014).

### Differentiation and Grouping Practices as Dimensions of Inclusive Teaching

Based on the educational needs of students, the didactic approach of differentiation (or differentiated instruction) aims at preparing teaching and learning contents in such a way that they are accessible and understandable for all students (Tomlinson, 2014; Suprayogi et al., 2017; Parsons et al., 2018). Differentiation as a didactic strategy can be implemented in different ways, for example in the form of collaboration and co-teaching, grouping, modification (of assessment, content, extent, instruction, learning environment, material, process, product, time frame), individual motivation and feedback, and personnel support of students (Eisenmann and Grimm(eds), 2016; Lindner and Schwab, 2020; Nusser and Gehrer, 2020). The variety of possible uses of differentiation enables teachers to respond individually to students’ characteristics (Tomlinson and Imbeau, 2010). As differentiation is a complex construct which demands high competence from teachers (Deunk et al., 2015; van Geel et al., 2019) they might struggle in practicing differentiated approaches and therefore, implement corresponding didactic techniques insufficiently (Groenez et al., 2018; Whitley et al., 2019; Gehrer and Nusser, 2020; Gheyssens et al., 2020).

Grouping practices can be categorized as a form of differentiation (Blatchford et al., 2003). While some students might work in pairs, others complete tasks on their own or in small groups (Buli-Holmberg and Jeyaprathaban, 2016). Furthermore, grouping practices can be considered as a feature of social classroom organization (Molbaek, 2018). “Groupings can be of different sizes and compositions and can vary in the amount of adult support they receive, the curricula and tasks they are given and the degree and quality of interaction between pupils” (Baines et al., 2003, p. 10; see also Blatchford et al., 2003; Kutnick et al., 2005). Taking into account the use of grouping strategies in the course of time and school development, studies on teaching and learning show in retrospect that frontal teaching, individual work and partner work were traditionally considered to be most effective and therefore were most frequently used by teachers due to their behavioral framework character (Götz et al., 2005). Terhart (2000) describes the teacher as responsible for methodological decision making but highlights that not only individual teacher preferences influence teacher’s decisions on grouping strategies. “The scope for methodological decision-making and action is limited, not only by conditions in the person of the teacher […] but also, and with a structurally resounding effect, by conditions that lie in the external, institutional arrangement of the teaching-learning process” (Terhart, 2000, p. 60). In principle, teachers have the possibility to implement different versions of grouping strategies adapted to the needs of their students and the appropriate didactic and methodological approaches of a lesson. Pioneer theoretical considerations on the relation of learning and organizational classroom settings highlight the importance of interaction between individuals and their environment, claiming that the learning environment (e.g., the classroom) can and should be used to the advantage of learning processes regarding individual learning conditions as well as competent and successful interactions between students, peers and teachers (Vygotsky, 1978; see also O’Donnell and King, 1999).

### Predictors of Inclusive Teaching Practices

According to previous research results, the implementation of cooperative grouping strategies correlates with teachers’ years of teaching experience. The greater the experience the more often teacher-controlled methodologies such as frontal teaching and independent single work are used (Saborit et al., 2016). Research on teaching experience and its impact on teachers’ use of differentiation as an inclusive practice shows contrasting results. On the one hand, there are results stating that novice teachers tend to try new innovative teaching trends, whereas well-experienced teachers are more likely to avoid trying out innovative methods and didactical and methodological approaches (Hargreaves, 2005). In contrast to this finding, other results suggest that teachers with a low number of years of experience reported significantly less implementation of differentiation than teachers with more work experience (Suprayogi et al., 2017; Schwab et al., 2019). Additionally, there are some studies that report no relation between years of teaching experience and the use of differentiation at all (McMillan, 2011; Lindner et al., 2019; Moosa and Shareefa, 2019).

With regard to demographic variables on teacher level, professional qualification and content-related teacher training are often considered to be positively influencing predictors for teachers’ use of inclusive teaching practices. Several studies state that there is a significant positive correlation between the knowledge of didactic constructs such as differentiated instruction and grouping practices and their scope of implementation and its usage in the classroom (McMillan, 2011; Brentnall, 2016; Park and Datnow, 2017). Moreover, the perceived quality and benefit of teacher trainings is positively associated with teachers’ implementation of differentiation and grouping practices (Hartwig and Schwabe, 2018). Considering studies that focus on the relationship between teaching practice and teachers’ gender, gender could not be found as possible predictor of teachers’ implementation of specific approaches (Opdenakker and van Damme, 2006; Saborit et al., 2016).

On class level, variables that might have impact on teachers’ practice encompass the class size, class composition as well as the educational setting (regular, inclusive, special class). Considering class size as possible predictor for teaching practices, numerous studies have come to the conclusion that there is an existing association between the two variables (Hattie, 2005; Blatchford, 2012, 2016). Results show that class size (the total number of students in one class) directly affects grouping practices regarding the number and sizes of individual groups when teachers organize their students into smaller groups (the higher the number of students in one class, the more smaller groups are formed; Blatchford et al., 2001; Blatchford and Russell, 2019). In the context of the current study, it seems interesting to investigate whether class composition variables (number of students with special educational needs, number of students with migration background, number of students with low SES) predict teachers’ implementation of differentiation and grouping practices. Regarding class composition, studies show that inclusive teaching practices, most notably differentiated teaching approaches, are practiced to a high degree when students with special educational needs (SEN) are a part of the class (Feyerer, 1998; Gebhardt et al., 2014). This result is also meaningful for a comparison of implemented teaching practices at the level of the educational setting. Studies showed that in integrative classes, inclusive teaching practices are more likely to be implemented than in regular classes (Feyerer, 1998; Gebhardt et al., 2014). In contrast, when constructing differentiation rather as individual teaching approach for single students than class-oriented strategy, results of a quantitative study show that neither the class size, the number of students with SEN nor the number of students with migration background could be found as predictors for teachers’ student-specific ratings of their inclusive teaching practices (Lindner et al., 2019).

### The Current Study

The current study comprises a secondary data analysis of data of the German National Educational Panel Study (NEPS; Blossfeld et al., 2011). The focus lies on the investigation whether there are any differences regarding the implementation of inclusive teaching practices on a didactic (differentiation) and organizational (grouping practices) level. Analyses of the NEPS data show that heterogeneity of class composition is not related to the extent to which differentiation measures are used (Gehrer and Nusser, 2020). In this context the question arises whether specific educational settings (special schools, inclusive classes, non-inclusive regular school classes) are decisive for the implementation of differentiated teaching methods. In line with this, three research questions as well as additional hypotheses are formulated:

(1) Are there differences regarding the use of differentiation as teaching strategy and the use of grouping practices between German classes at special schools, inclusive classes, and regular school classes?

(2) Are there latent profiles to be identified based on the use of grouping practices? If there are latent profiles, are some of them more common in specific educational settings?

Against the background outlined above, the hypothesis is put forward that among teachers of different educational settings differences in the usage of differentiation and grouping practices can be found (e.g., Feyerer, 1998; Gebhardt et al., 2014). In addition to the comparison of inclusive versus regular classes, we extend the comparison to special schools as third type of educational setting.

(3) Do the educational setting, contextual variables of class composition and individual teacher characteristics contribute as predictors to differentiation and grouping practices?

The formulation of hypotheses regarding possible predictors seems challenging, as results of individual studies contrast specific variables and their influence on teachers’ implementation of differentiation and grouping practices.

## Materials and Methods

### Sample

This study draws on the data of the NEPS in Germany (Blossfeld et al., 2011). The longitudinal study NEPS including six different starting cohorts aims at investigating educational trajectories and competence development across the life course. Initial samples are representative for a specific age group of targets (e.g., fifth graders, ninth grader, first year university students; Aßmann et al., 2011). To gain a broader picture of the situation of participants and to address context effects and influences of learning environments also parents, educators, teachers, and principals are invited to participate in the study when possible and meaningful. This paper uses data of starting cohort 3 located in secondary school^1^. Measurement occasions comprise surveys within schools to administer competence tests and questionnaires for the students themselves. Trained administrators visit the schools to guarantee comparable and standardized procedures. They also hand out paper-based questionnaires to the teachers of the participating classes including home-room teachers and teachers for the subject German. Participation is voluntary and includes informant consent by all participants.

This paper draws primarily on data of teachers who are teaching German classes in different school settings in secondary school (grade 5–8). Overall, data of the first four measurement occasions are used. Information from home-room teachers are used to gain information on class composition in more detail. Based on school information (special school or mainstream school), individual information on SEN status of participating students and on information on the proportion of students with SEN in the area of learning (SEN-L) within class, teachers of the analytic sample are categorized as either teaching a special class (defined as being taught in special schools), an inclusive class (defined as including students with SEN-L in mainstream schools) or a non-inclusive regular class (no students with SEN at mainstream school). Over the course of four measurement points, 4191 German classes can be identified. Due to unit-non-response and failure of reliable categorization into the three groups, the analytic sample of educators teaching German comprises *N* = 1034 (see Table 1 for sample characteristics).

**TABLE 1:** Teacher characteristics and classroom composition.

*N* = *1034 teachers teaching German in 5th grade*	*Female*	*Migration background*	*Average years of teaching experience*	*Need for further training*	*Class size (number of students)*	*Percentage female students*	*Percentage of migrant students*	*Percentage of students from academic household*
*Non-inclusive classes in regular schools; N = *755**	72.40%	8.51%	15.27 (11.27)	83.50%	25.34 (4.23)	47.95%	24.74%	23.72%
*Inclusive classes in regular school; N = *89**	78.48%	2.08%	16.80 (15.87)	81.82%	20.89 (4.31)	45.33%	31.42%	14.87%
*Special schools for SEN-L; N = *190**	84.57%	3.33%	15.70 (11.76)	67.24%	11.99 (2.06)	42.82%	28.50%	1.43%

*Standard deviation reported in brackets.*

The majority of 755 teachers teach in non-inclusive regular school classes. Of those, about 73% are female, almost 9% have a migration background (meaning at least one parent being born abroad) and the average years of teaching experience amounts to 15.27 years (*SD* = 11.27). Over 80% state the need for further teacher training. 89 teachers work in inclusive classes in regular schools (at least one student with SEN-L in the class). Within this sample group, 78% are female, 2% have a migration background, the average years of teaching experience are 16.80 years (*SD* = 15.87) and again over 80% state the need of further teacher training. Hundred and ninety teachers of the sample teach in special schools with special emphasize on SEN-L. The majority of teachers (85%) in these classes are female, around 3% have a migration background. The average amount of years of teaching experience is 15.70. Almost 68% state the need for further teacher training. The years of teacher experience range from 0 to 42 years for the whole sample.

### Classroom Settings

#### Regular Classroom Setting

The regular classroom setting encompasses classes in regular school without students diagnosed as having SEN-L. The average number of students in these classes is 25.34. About 48% of students are female, almost a quarter of the students have a migration background (operationalized through the presence of at least one parent born abroad) and about 24% have parents with an university degree.

#### Inclusive Classroom Setting

The investigated inclusive classrooms can be defined as classrooms in which students without SEN and students with SEN-L are taught together. The diagnosis of special educational needs in learning was chosen as the focus, as it can be assumed that students with SEN-L need special help in learning and competence acquisition as well as experience difficult access to learning content. In these classes the average number of students is 20.89. About 45% of students are female, almost one third of students have at least one parent born abroad and around 15% parents with an university degree.

#### Special Classroom Setting

Within the participating special schools, students with special educational needs in learning are taught. The selection of these classes enables the comparison between inclusive setting and special schools with focusing on teaching students with SEN-L. The average number of students is about half of the other classes (11.99 students on average). Less than half of students in special classes are female (43%), 28.5% have a migration background and only few parents of (1.43%) of students have parents with an university degree.

### Instruments

Data were collected using a paper-based questionnaires given out to teachers for the subject German. A correlation matrix of all variables can be found in the Appendix C.

#### Differentiation as Didactic Dimension

The four items considering differentiated instruction were developed following the work of Ditton (2000). In this context, differentiation as a construct is operationalized through teachers’ consideration of students’ academic performance when designing tasks. It comprises qualitative and quantitative forms of differentiation, e.g., homework of varying difficulty depending on their performance (quality), specific additional tasks for further comprehension (quantitative) (see Appendix A). Participants were asked to respond using a 5-point Likert scale ranging from 1 = not at all true to 5 = completely true. The one-dimensional factor structure of the 4-item scale has already been confirmed across all measurement points (Gehrer and Nusser, 2020). Measurement invariance between teachers in special, inclusive, and regular schools was also investigated and proven. Thus, comparable analysis are eligible.

#### Grouping Practices as Organizational Dimension

The scale on teachers’ use of grouping practices during teaching consists of seven items including the work with small groups, partner work, discussion groups, students as tutors in the sense of peer tutoring, joint discussion between teacher and students, individual work, and frontal teaching (see Appendix B). Teachers were asked to rate the amount of usage of the specific grouping practices on a 6-point Likert scale encompassing 1 = never, 2 = one to two times a school year, 3 = every few months, 4 = every 2–4 weeks, 5 = once a week, 6 = (nearly) every lesson. Different forms of grouping practices and class room organization are not one-dimensional (e.g., DeVries et al., 2020 for a three-factor-solution). However, based on the weak reliability of the three-factor solution, the current article focuses on single indicators and patterns of use of grouping practices.

#### Predictor Variables

With respect to the context of the classroom primarily structural variables of class composition are considered as predictors. The raw number of students within a class laying the ground for grouping practices (ranging from 5 to 39 students). Moreover, the percentage of female students, students with a migrant background (at least one parent born abroad), and students living in an academic household (at least one parent having received an academic degree) are entered into the model. The following teacher characteristics are additionally considered. The binary variable migrant background of teachers is operationalized through the presence of at least one parent being born abroad (0 = no migrant background; 1 = migrant background). With respect to the need for further teacher training, topics are included that deal with the focal points of teaching students with special educational needs in learning and inclusive teaching practices such as differentiation and individualization. If teachers indicated the need of further training in one of the aforementioned areas the binary variable is coded as 1.

### Analyses

The initial aim of the current paper deals with the question whether there are any differences in teachers’ perceived implementation of differentiation and grouping strategies in different educational settings. To this end, we conducted one-way ANOVAs followed by the Tukey HSD *post hoc* test. All descriptive analysis are conducted in R (R Core Team, 2013).

Considering the question about differences regarding regular, inclusive and special teachers’ implementation of grouping practices, latent profile analyses (LPA) were calculated. LPA allows to examine the distribution of teacher groups and patterns in the implementation of grouping practices (Muthén and Muthén, 2017). Regarding the limited evidence-based knowledge on the relations of teachers’ use of inclusive teaching practices and the educational setting, LPA are potentially informative. Therefore, the classification analysis is calculated to form latently unknown teaching profiles regarding the use of grouping practices based on manifest variables (items of grouping practices scale). LPA is considered to be a worthwhile approach to identify potential teaching styles. This method allows to identify “unobserved subgroups (classes) within the sample by maximizing the homogeneity within subgroups while maximizing the heterogeneity between subgroups” (Mammadov et al., 2016; for advantages of LPA see: Muthén and Muthén, 2000; Eshghi et al., 2011).

LPA is a person-centered approach that reveals patterns – in our case – of teaching practices that allows to further investigate specific teaching styles that do not represent a one-dimensional factor but rather integrates divers aspects of teaching.

To examine predictors for teachers’ use of inclusive teaching practice (implementation of differentiation and profiles of grouping practices), predictors on class level (educational setting, class size, class composition) and teacher level (gender, migration background, teaching experience, perceived need for further teacher training) linear and multinomial regression analysis are conducted. The LPA as well as the regression analysis are conducted with Mplus 8 (Muthén and Muthén, 2017). Missing values are handled using the full information maximum likelihood approach (FIML).

## Results

### Descriptive Results

The results (see Table 2) to research question 1 (see section “The Current Study”) regarding whether there are differences in the use of differentiation as teaching strategy and the use of grouping practices between German classes at special schools, inclusive classes, and regular school classes show that differentiation is used significantly more often in inclusive classes than in regular classes where no student with SEN-L is placed. Additionally, teachers in special schools report a significant higher implementation of differentiation than teachers in inclusive classes.

**TABLE 2:** Descriptive results on use of differentiation and grouping practices.

Teachers’ use of grouping practices					

	Mean scores (*SD*)				ANOVA
	
	Total sample	Regular class	Inclusive class	Special class	
Differentiation	3.27 (0.79)	3.08 (0.71)	3.39 (0.74)	3.98 (0.71)	*F*(2,1031) = 120.71, *p* < 0.001
Small groups	4.38 (1.04)	4.37 (1.05)	4.44 (1.08)	4.41 (1.00)	*F*(2,1031) = 0.23, *p* = 0.80
Partner work	5.20 (0.87)	5.30 (0.84)	5.03 (0.93)	4.84 (0.83)	*F*(2,1031) = 24.28, *p* < 0.001
Discussion groups	4.15 (1.15)	4.17 (1.17)	3.91 (1.02)	4.17 (1.13)	*F*(2,1031) = 2.04, *p* = 0.13
Peer tutoring	2.75 (1.46)	2.71 (1.42)	2.98 (1.53)	2.71 (1.55)	*F*(2,1031) = 1.23, *p* = 0.29
Joint discussion between teacher and students	4.78 (1.08)	4.82 (1.03)	4.67 (1.11)	4.64 (1.23)	*F*(2,1031) = 2.54, *p* = 0.08
Individual work	5.09 (0.85)	5.02 (0.85)	5.19 (0.80)	5.30 (0.87)	*F*(2,1031) = 8.94, *p* < 0.001
Frontal teaching	5.71 (0.55)	5.70 (0.56)	5.75 (0.51)	5.76 (0.51)	*F*(2,1031) = 1.26, *p* = 0.28

A one-way ANOVA revealed that there was a statistically significant difference in mean of differentiation between the three classroom settings, *F*(2,1031) = 120.71, *p* < 0.001.

Tukey *post hoc* test for multiple comparisons revealed higher scores for differentiated instruction in special classes compared to inclusive classes (0.59, 95%-CI[0.37, 0.80]) and compared to regular classes (0.89, 95%-CI[0.75, 1.03]). Moreover, multiple comparisons found that the score of differentiated instruction is higher in inclusive classes compared to regular classes (0.31, 95%-CI[0.12, 0.50]).

Regarding teachers’ use of grouping strategies, results indicate that in general teachers in different educational settings have a similar frequency in implementation (see Table 2).

Overall, significant differences in the use of teaching strategies are found with respect to the implementation of partner work and individual work. Tukey *post hoc* analysis revealed lower frequency for partner work in special classes (–0.46, 95%-CI[–0.62, –0.30]) and inclusive classes (–0.27, 95%-CI[–0.49, –0.05]) compared to regular classes. No difference was found between special and inclusive classes. On the contrary, individual work is used quite more often at special schools compared to regular classes (0.27, 95%-CI[0.12, 0.44]).

### Latent Profile Analysis Regarding Teachers’ Use of Grouping Practices

To answer research question 2 (see section “The Current Study”) and to examine the distribution of teachers’ didactic style considering the implementation of grouping practices, latent profile analysis was calculated. Analyses with up to five profiles are conducted to select a number of profiles fitting the data best. Based on the information criterion AIC and BIC profile solution with more profiles seems to fit better to the data. But group sizes get too small to interpret results in a more general perspective (smallest group size of 3% for the 5-profile-solution). Investigating the indices of entropy (quality of profile classification) and average latent class probability of each of the profiles (Celeux and Soromenho, 1996), the 3-profile-solution should be preferred (see Table 3). Considering theoretical and empirical results jointly, the solution including three profiles will be investigated more closely.

**TABLE 3:** Calculation of meaningful profiles based on items of grouping practices scale.

*N* = 1034	AIC	BIC	Entropy	Lowest average latent class probability	Smallest group size
(1) Profile	20002	20071	1.00	1.00	100%
(2) Profiles	19413	19522	0.788	0.91	25%
(3) Profiles	18741	18889	0.857	0.87	20%
(4) Profiles	18645	18833	0.834	0.80	11%
(5) Profiles	16430	16658	0.855	0.80	3%

**FIGURE 1:**
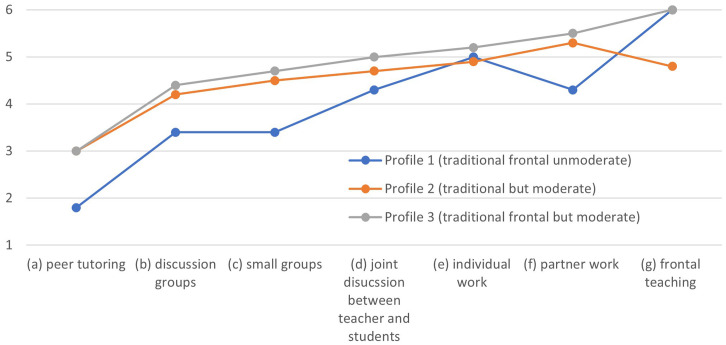
Three-profile solution, *x*-axis (sorted by frequency).

The three-profile solution (see Figure 1) shows that the profiles not only differ in the frequency of use of grouping but also in the individual focus on the use of specific strategies. Profile 3 shows the most intensive use of grouping practices with peer tutoring as the least used practice and frontal teaching the most used one (chronological order from a to g). Profile 3 reflects a rather traditional use of teaching methods (Götz et al., 2005). Due to the distribution of grouping practices, this profile is referred to as the traditional frontal but moderate profile. Particularly frequently implemented strategies are characterized by a focus on the role and didactic authority of the teacher (frontal teaching, partner work, individual work). However, the profile is called traditional frontal but moderate as, although the most frequently used practice is frontal teaching, all rated grouping practices are used at least every few months. Profile 2 (traditional but moderate profile) indicates a similar order of focal points starting with peer tutoring as the least used practice and partner work the most implemented one (order of extent of use of grouping practices: b, c, a, d, e, f, g). The distribution of group practices in profile 2 is similar to the characteristics of profile 3. The order of the most frequently used methods differs only with regard to frontal teaching, which in profile 2 moves from the first to the third place and thus ranks behind partner work and individual work. Profile 1 (traditional frontal unmoderate) differs most clearly from the other two profiles. It differs both in the frequency of use and the individual focus when it comes to the implementation of grouping practice (order of extent of use of grouping practices: a, c, b/d, e/f, g). In contrast to the other two profiles, the average rating tendency of the individual grouping strategies ranges from never/once or twice a year (peer tutoring) to (almost) every lesson (frontal teaching). Therefore, this profile is referred to as the traditional frontal unmoderated profile. It should be emphasized that we cannot contrast the three profiles, which are defined as traditional in their basic form, on a modern/alternative profile. All three teaching styles remain traditional in their teaching and learning characteristic.

The calculation of the three-profile solution shows that profile 3 (traditional frontal but moderate), which indicates a high and frequent extent of use of grouping practices is the most representative profile for all three educational settings (regular class 55.36%, inclusive class 58.43%, special class 55.26%; see Table 4). This profile implies that the members of profile 3 have moderate to high probability for all items [frequency 3 = every few months (3.032 ± 0.106) to 6 = nearly, every day (6.000 ± 0.000)]. In regular classes, profile 1 (traditional frontal unmoderate) is less represented than profile 2 (traditional but moderate), whereas a contrary picture emerges regarding special classes, where profile 1 is more often represented than profile 2 (traditional but moderate).

**TABLE 4:** Breakdown 3-profile solution.

*n*_*profiles*_	Percentage of total sample	Percentage regular class	Percentage inclusive class	Percentage special class
Profile 1 (*n* = 205)	19.83%	18.68%	20.22%	24.21%
Profile 2 (*n* = 254)	24.56%	25.96%	21.35%	20.53%
Profile 3 (*n* = 575)	55.61%	55.36%	58.43%	55.26%
Total sample (*n* = 1034)	100%	100%	100%	100%

*Bold values display statistically significant values.*

### Predictors for Teachers’ Use of Differentiation and Grouping Practices in Regular, Inclusive and Special Classes

#### Prediction of Teachers’ Use of Differentiation

A linear regression model was calculated to explain teachers’ implementation of differentiation by using predictors on class and teacher level answering research question 3. In a first model, only predictors regarding the educational setting are included. The results (see Table 5) show that teachers of special classes tend to use differentiated teaching approaches more often than teachers of regular classes (model 1: β = 0.53, *p* < 0.05). This result remains stable throughout the different models (model 4: β = 0.41, *p* < 0.05). On context level, two predictors are even when controlling for individual characteristics on teacher level statistically significant for teachers’ use of differentiation: class size (model 4: β = –0.18, *p* < 0.05) and the number of students with migration background in class (β = 0.10, *p* < 0.05). Regarding class size, the results show that the higher the total number of students in one class the less teachers tend to implement differentiated teaching practices. The number of students with migration background has a positive effect on teachers’ implementation of differentiation. The more students with migration background are in a class, the more teachers use differentiation. On teacher level, none of the selected independent variables (e.g., years of experience, need for further teacher training) could predict teachers’ use of differentiation.

**TABLE 5:** Model results for differentiation (linear regression).

*N* = 1034	Model 1	Model 2	Model 3	Model 4
*Setting*				
Inclusive classes (compared to regular classes)	–0.01 (0.06)	0.03 (0.06)	–0.001 (0.06)	0.03 (0.06)
**Special classes (compared to regular classes)**	0.53* (0.06)	0.41* (0.07)	0.39* (0.05)	0.41* (0.07)
*Context variables*				
**Class size**		–0.18* (0.06)		–0.18* (0.06)
Number of female students		0.06 (0.04)		0.06 (0.04)
**Number of students with migration background**		0.10* (0.04)		0.10* (0.05)
Number of students with one parent with academic degree		–0.05 (0.08)		–0.06 (0.08)
*Teacher variables*				
Gender (0 = male; 1 = female)			0.08 (0.07)	0.02 (0.04)
Migration background (0 = no; 1 = yes)			–0.15 (0.17)	–0.06 (0.05)
Years of teaching experience			–0.01 (0.03)	–0.01 (0.09)
Stated need for further teacher training			0.14 (0.09)	0.05 (0.04)

aBIC	11333	17601	13757	20600

*Latent modeling of dependent variable differentiation with four items. Bold values display statistically significant values.*

#### Prediction of Teachers’ Profile in Using Grouping Practices

A multinomial logistic regression was used to predict teachers’ implementation style of grouping strategies. Membership to the identified profiles are used as nominal dependent variable. Models include several independent possible predictors on class and teacher level. First, a model with only the educational setting as predictor is calculated (see Table 6). For every model the reference is the probability of tendency to implement grouping practices according to the most common profile 3 (traditional frontal but moderate profile). When explaining teachers’ use of grouping strategies on the basis of the educational setting, a statistically significant difference can be found regarding the comparison of regular and special class teachers. The probability that the professional actions regarding teaching practices of special teachers match profile 2 (traditional but moderate profile) is significantly lower than for regular teachers (β = –1.60, *p* < 0.05). Second, in addition to the educational setting, context variables as possible predictors for teachers’ implementation of teaching practices are investigated. This model indicates that the educational setting is no longer a statistically significant predictor for teachers’ use of practices, but the class size as context variable. The results show that the more students in a teachers’ class are, the more likely the teacher is to implement grouping practices according to profile 2 (traditional but moderate profile) compared to profile 3 (traditional balance frontal profile; β = 0.73, *p* < 0.05). Another significant result in model 2 refers to the number of students with at least one parent with an university degree in class. The higher the amount of these students the less likely teachers are to implement grouping practices in the style of profile 1 (traditional frontal unmoderate profile) in comparison to profile 3 (traditional frontal but moderate profile; β = –1.08, *p* < 0.05). When checking the educational setting and teacher variables as possible predictors in the third model, no correlation between the setting and the established use of grouping practices could be found. However, teacher characteristics correlate with teachers’ implementation of grouping practices. Regarding teachers’ years of teaching experience, two significant results can be reported: The more years of teaching experience teachers have, the more likely they are to implement grouping strategies according to profile 1 (traditional frontal unmoderate profile) compared to profile 3 (traditional frontal but moderate profile; β = 0.75, *p* < 0.05). In contrast, the more experienced teachers are, the less likely they are to rather practice grouping strategies in the style of profile 2 (traditional but moderate profile) than profile 3 (traditional frontal but moderate profile; β = –0.89, *p* < 0.05), which means that the use of frontal teaching becomes higher with increasing years of teaching experience. Considering teachers’ perceived need of further teacher training on topics related to heterogeneity, the following result can be reported regarding model 3: Teachers are more likely to correspond with profile 3 (traditional frontal but moderate profile) than profile 1, if need for further training is stated.

**TABLE 6:** Model results for grouping practices used with a 3-profile solution (multinomial regression).

*N* = 1034	Model 1		Model 2		Model 3		Model 4	
				
	Profile 1	Profile 2	Profile 1	Profile 2	Profile 1	Profile 2	Profile 1	Profile 2
*Setting*								
Inclusive classes (compared to regular classes)	0.32 (1.46)	0.84 (1.24)	0.09 (0.46)	0.30 (0.53)	0.07 (0.39)	0.19 (0.33)	0.05 (0.31)	0.15 (0.28)
**Special classes (compared to regular classes)**	0.72 (1.37)	–1.60* (0.70)	0.21 (0.54)	–0.34 (0.63)	0.17 (0.40)	–0.41 (0.34)	0.09 (0.36)	–0.20 (0.33)
*Context variables*								
**Class size**			0.47 (0.34)	0.73* (0.34)			0.21 (0.33)	0.49 (0.28)
**Number of female students**			–0.16 (0.30)	0.44* (0.32)			–0.15 (0.20)	0.41* (0.18)
Number of students with migration background			–0.32 (0.34)	0.10 (0.39)			–0.23 (0.25)	–0.09 (0.22)
**Number of students with one parent with academic degree**			–1.08* (0.16)	–0.83* (0.37)			–0.58 (0.34)	–0.59 (0.32)
*Teacher variables*								
Gender (0 = male; 1 = female)					–0.18 (0.25)	–0.03 (0.20)	–0.21 (0.20)	–0.07 (0.19)
Migration background (0 = no; 1 = yes)					–0.08 (0.35)	–0.37 (0.25)	–0.05 (0.27)	–0.34 (0.23)
**Years of teaching experience**					0.75* (0.26)	–0.89* (0.15)	0.66* (0.31)	–0.79* (0.20)
**Stated need for further teacher training**					–0.61* (0.27)	–0.31 (0.26)	–0.51* (0.21)	–0.27 (0.23)

aBIC	2069		8370		4497		10826	

*Report are of standardized coefficients. Standard errors in brackets. Reference is always profile 3.*

*p < 0.05. Bold values display statistically significant values.

Regarding the results in the fourth model combing all variables, the same trend for years of teaching experience and stated need for further teacher training can be reported. Additionally, the number of female students is a positive predictor for teacher probability to teach in the style of profile 2 (traditional but moderate profile) rather than profile 3 (traditional frontal but moderate profile; β = 0.41, *p* < 0.05). In model 4 on the contrary, the predictors educational setting, class size, and number of students with one parent with academic degree show no longer a statistically significant correlation in contribution to the explanation of teachers’ profile regarding implementing grouping practices.

## Discussion

This study aimed to investigate German teachers’ implementation of differentiation and grouping practices in inclusive classes, regular classes as well as special classes. An in-depth investigation of inclusive teaching practices within these different educational settings (regular, inclusive, and special education setting) seems insightful to understand the link between students’ diversity, special educational needs in learning (SEN-L) and teachers’ daily teaching practices. Until now, a study with this focus using a large-scale longitudinal sample was lacking in the German-speaking area.

The main findings of this study are that teachers in special school classes tend to use more often differentiation than teachers in inclusive and non-inclusive classes of regular schools. Furthermore, few differences were found regarding teachers’ use of within-class grouping strategies between all three selected classroom settings (regular class, inclusive class, special class). The latent profile analysis (LPA) revealed three types of teachers’ didactic styles regarding their implementation of grouping strategies. Teachers’ affiliation to a profile depends on students’ gender, teachers’ years of teaching experience, and their self-identified need for further teacher training.

Focusing on the frequency of teachers’ use of differentiation, the results of the present study report that in special classes differentiation is practiced the most, while in inclusive classes teachers differentiate a bit less and in regular classes the lowest frequency of differentiation was found. Regarding single items, partner work is less implemented in special and inclusive classes than in regular classes, whereas individual work is used more often in the two settings compared to regular class settings. One possible explanation would be that teachers of different educational settings have undergone specific teacher trainings based on different curricula (Gebhardt et al., 2014). Another explanation might be that the classroom composition differs when comparing the three types of settings. Whereas in special classes, only students with special educational needs are together, in inclusive classes students with and without SEN-L and moreover, in regular classes only students without SEN-L are taught together. Therefore, maybe the diagnosis as having SEN-L triggers teachers’ awareness of the need of differentiated and personalized instruction as there is a label or category that they can refer to as prerequisite for adapted teaching methods (Gebhardt et al., 2014). With regards to the differences in teachers’ ratings of single items (lower partner work in special and inclusive classes, but higher individual work), similar explanations might justify these results. Along with a greater awareness of the need for individualized tasks, which is rooted in special teacher training, one possible explanation might be that teachers in special and inclusive classes are more responsive to the individual educational needs of their students and therefore use more forms of individualized work than partner work. In line with this, results from regression analysis indicated that teachers’ use of differentiation is higher in special classes compared to regular classes even when controlling for variables regarding class composition (e.g., class size, number of female students in class) and individual teacher characteristics (e.g., gender, migration background). In addition, the class size has a negative influence on the use of differentiation. The higher the class size, the less teachers use differentiation practices. This result is reflected in the consideration of the educational settings. In special educational settings (where the use of differentiation was highest), the lowest number of students on average is in one class. In this context, the interplay of class size, educational setting and use of differentiation can be observed. From a student-centered perspective, the result seems surprising, since a higher number of students also implies a greater variety of individual needs that require teachers’ didactic consideration and action. Possible reasons for this result could be excessive demands, too few material and personal resources available, the need for intensive preparation time as well as the complexity of the implementation and evaluation of differentiated practices.

Teachers’ variables (gender, migration background etc.) do not significantly predict the implementation of differentiation. However, other variables like e.g., teachers’ constructivist beliefs (see Pozas et al., 2020) or teachers attitudes toward inclusive education (see e.g., Schwab and Alnahdi, 2020) might be underlying factors which are not investigated within the present study.

Focusing on grouping strategies, another picture appears. There are no differences found between inclusive and special classes. Considering the three identified profiles (three-profiles solution) regarding teachers’ implementation of grouping practices, their tendency to use traditional strategies is particularly striking. In general, differences could be identified on two different levels: balance in the use of strategies and frequency. Overall, the order (ranked by frequency) was nearly the same for all profiles, starting with traditional practices such as frontal teaching, partner work, individual work and joint discussions between teachers and students with the highest frequency within all three profiles. In contrast, small group work, student discussion groups and peer tutoring sequences were used less often. To take up once again the term traditional, profile 1 was referred to as the traditional frontal unmoderate profile, whereas profile 2 was described as traditional balanced profile and profile 3 (which applies to over 50 percent of the participating teachers) as traditional balanced frontal profile, as they mainly differ according to the ranking of frontal teaching practices. What is obvious is that each profile has traditional characteristics as explained above. Profile 2 and 3 are different in their use of frontal teaching as profile 3 encompasses a higher usage of this strategy. Nevertheless, the implementation of every form of grouping practice can be considered as relatively balanced. Profile 1 also has traditional traits and almost the same arrangement of single grouping practices as the other two profiles. However, this profile shows a comparatively imbalance of specific grouping practices regarding their implementation.

Possible explanations for teachers’ high frequently use of traditional practices may be due to specific characteristics attributed to these strategies. It is assumed that traditional practices are mainly teacher-led in terms of classroom management and freedom in working (Terhart, 2000; Götz et al., 2005). Therefore, with less students’ participation and self-regulation, controlling and keeping an overview may be easier for the teachers. Self-directed, participatory strategies may be more difficult to handle for teachers and require accurate, intensive preparation, implementation, and evaluation (Emanuelsson and Sahlström, 2008).

Further results from regression analysis indicate (including class setting, class variables as well as teacher variables) that teaching practices of teachers with longer teaching experience are more often assigned to profile 1 (most traditional frontal unmoderated profile) compared to profile 3 (traditional frontal but moderate). Additionally, teachers who feel the need for further teacher training more often correspond with profile 1 than with profile 3. This is particularly interesting, as profile 1 and profile 3 mainly differ in terms of a balanced use of practices. Profile 1 shows clearer differences in the frequency of use of traditional (higher extent) and alternative methods (lower extent) in teaching. Therefore, it can be assumed that using frontal teaching and other traditional strategies (individual work, partner work, joint discussion) seem to be established practices, which are used by less experienced teachers who feel the need for higher training and high experienced teachers who have received traditional teacher training that goes back some time. This should and can easily be addressed in further in- and pre-service teacher trainings by introducing new methods to teachers and give them space to get familiar with these practices. Another possibility to ensure a variety in the use of methods is the formation of team-teaching groups of experienced/specifically trained and non-experienced teachers to enable exchange and alternation in practice (Anthony et al., 2019). In addition to that, the provision of additional support for teachers and students such as teaching assistants or temporary/permanent team teaching should be reviewed. In this context, not only teaching assistants’ or additional teachers’ frequency of their presence and work in the classroom should be investigated, but also their role when being present which can have an significant impact on teachers’ pedagogical decision making and students’ perception and experience of classroom processes (Radford et al., 2015; Wren, 2017; Bosanquet and Radford, 2019). Interestingly, in classes with a higher number of female students, profile 2 is more common compared to profile 3. This indicated that the more boys are in class, the higher is the probability that teachers use frontal teaching as most frequent practice. This result could indicate that teachers are convinced that female students show a lower level of deviant class behavior and more prosocial behavior (see e.g., Lansford et al., 2012) and therefore teachers are more likely to use a less teacher-centered teaching style.

Addressing the sample of this study, descriptive results confirm that the classroom compositions of the three settings was not evenly distributed as e.g., the socio-economic capital of students is much higher in regular classes compared to inclusive ones and lowest in special classes. The fact that specific students (e.g., low socio-economic background, low educational degree of parents, boys) are more likely to have a diagnosis of SEN underpins a well-discussed reliability in Germany (e.g., Labsch et al., 2021). Contrary, the condition that inclusive classes are cumulating at-risk students (e.g., students with migration background, lower socio-economic status) was less often addressed in research (George and Schwab, 2019). This might be an important aspect when comparing outcomes of inclusive and regular classes and underpins the necessity for studies to check classroom composition variables when comparing different educational settings. In the context of the present study, the consideration of the class composition from teachers’ perspective seems to shed light to the necessity of an appropriate selection of didactic and pedagogical measures. Furthermore, the question arises to what extent only a diagnosed special educational status of students should be decisive for differentiation and individualization, or rather a variety of individual characteristics of every student regardless a diagnosis of having special educational needs should be considered by teachers.

## Limitations

One limitation of this study is part of its design – only teacher ratings of teaching and grouping strategies were available. As teachers might be aware that they should use a rather broad range of inclusive teaching strategies they might have given rather biased answers. E.g., Lindner et al. (2019) already showed that students experience less inclusive teaching strategies compared to teachers’ perceptions. Therefore, for future research it would be interesting to combine several methodological approaches, e.g., also do classroom observations and use the true scores (e.g., counting the minutes how long specific strategies are used). In addition, as Gehrer and Nusser (2020) demand, not only the quantity but also the quality of inclusive teaching practice should be ascertained, especially in the area of differentiated teaching measures and materials.

Considering the definition of inclusive classes with regards to the sample of the study, it should be noted that this term is based on school policy framework. In Germany, the placement of at least one student with SEN in a regular school class leads to that class being referred to as an inclusive class. However, the placement is not necessarily associated with a changed orientation of the pedagogical classroom and teaching culture. Therefore, this is a political definition of inclusion, which is not to be equated with the actual understanding of inclusion underlying the article (e.g., Ainscow and Messiou, 2018; Schwab, 2019; United Nations Educational Scientific and Cultural Organization, 2020). Moreover, the results are only representing a sample of secondary teachers teaching German. A study (Baines et al., 2003) on grouping practices in primary and secondary schools shows that students on secondary education levels are less likely to work individuated, but more likely to engage with peers within interactive tasks than on primary school levels. Additionally, results show that teachers of secondary classes are more likely to alternate forms of grouping practices regularly (Baines et al., 2003). Further, since the study was limited to already collected data (NEPS, see e.g., Blossfeld et al., 2011) in the sense of a secondary analysis, it was not possible to adjust or add items that would have been helpful in understanding teachers’ underlying interpretation of differentiation and grouping practices. What seems desirable in this context, is the investigation of teachers’ professional reasons for pedagogical decisions, e.g., criteria for group composition, reasons for implementing specific grouping practices and the implementation of other forms of differentiation that go beyond the operationalization through student performance. In the context of the predictor variables, several limitations have to be addressed. Education inequalities and professional teaching decisions due to students’ migration background can often be traced back to linguistic diversity and therefore, it would make sense to operationalize migration background with regards to students first language (Kemper, 2010). Still, within this study students’ migration background is defined by the place of birth of the student itself and/or their parents and not by their first language. That way, also those students who may not be first-generation migrants (and who may have German as their first language in the second and third generations), but who are associated with migration by teachers are included. This approach allows to take teachers’ perceptions of their class into account. Literature shows that teachers’ subjective perception of students’ migration background (e.g., based on external characteristics, name, etc.; see e.g., Tobisch and Dresel, 2017; Bonefeld and Dickhäuser, 2018; Huber et al., 2018) also influences their pedagogical and diagnostic competencies. Additionally, with regards to the NEPS data and the current research interest, the definition of migration background operationalized through students’ first or family language would lead to a very small sample size which would make it impossible to calculate certain analysis. Thus, the operationalization via the country of birth of a parent is more meaningful in terms of analytical strength. Further research could investigate whether the use of different operationalization of migration background has an impact on the outcomes regarding teachers use of differentiation and grouping strategies as they might adapt their teaching differently when it comes to students with diverse first languages. In this context, whether one parent was born abroad (Destatis-Statistisches Bundesamt, 2021), does not directly give information on students first language at all. In addition, teachers’ visual perception of migration background resulting in students’ name, visual appearance and parental contact as attitude variable of the teacher could also lead to changed teaching decisions which should be considered within future research.

Regarding the predictor class size, it has to be mentioned that there are generally fewer students in special school classes than in regular school classes (regardless of whether inclusive or non-inclusive classes). Therefore, in the context of the study, class size cannot be treated as independent variable but rather depends on school track (special or regular school). Regarding school tracks as a predictor variable for differentiation and grouping strategies, a paper of Pozas et al. (2020) shows a very similar overall response pattern for German teachers’ use of differentiated teaching methods across school tracks. The authors also used NEPS data of teachers of SC3 in German and Math classes. In line with the results of the paper of Pozas et al. (2020), which presented view differences with a very small effect size in teachers’ use of differentiation regarding school tracks as predictor, the variable was not included in the current study. Nevertheless, further research could examine the influence of the school track on teachers’ use of grouping practices. Additionally, for further research it seems interesting to investigate whether there is a differential use of differentiation and grouping strategies within the selected classroom settings based on subject matter (e.g., comparison of German lessons and Math lessons; see also DeVries et al., 2020).

## Conclusion

Despite the mentioned limitations, results of this study can be used in educational policy (e.g., ensuring that there are not specific students overrepresented in some classes) as well as in teacher training (e.g., ensuring greater diversity management competencies). The current analytic approach can be considered as person-centered since various information on class composition and individual teacher characteristics have been included that have been found to be associated with teachers’ implementation of differentiated instruction. By considering the role of teachers and classroom composition, a differentiated view is taken. The current study encompasses a broad approach by investigating the role of teachers, classroom composition as well as a divers repertoire of teaching methods and examining potential teaching styles. The results enable a reflective examination of teaching methods, and the question of how inclusive teaching practices can be used in everyday school-life in a simple, resource-saving, yet target-oriented and effective manner, regardless of independent factors on teacher and context level.

## Data Availability Statement

Publicly available datasets were analyzed in this study. This data can be found here: https://www.neps-data.de/.

## Ethics Statement

The NEPS study is conducted under the supervision of the German Federal Commissioner for Data Protection and Freedom of Information (BfDI) and in coordination with the German Standing Conference of the Ministers of Education and Cultural Affairs (KMK) and – in the case of surveys in schools – the Educational Ministries of the respective Federal States. All data collection procedures, instruments and documents were checked by the data protection unit of the Leibniz Institute for Educational Trajectories (LIfBi). The necessary steps are taken to protect participants’ confidentiality according to national and international regulations of data security. Participation in the NEPS study is voluntary and based on the informed consent of participants. This consent to participate in the NEPS study can be revoked at any time.

## Author Contributions

K-TL: conceptualizing and design, literature research, theoretical foundation of the manuscript, consolidation of all ideas, writing of introduction, discussion and conclusion, revision and adjustment regarding ideas, and comments of all authors. LN and KG: conceptualizing and design, performance of analysis, writing of results, and comments on overall manuscript. SS: conceptualizing and design, writing of discussion, and comments on overall manuscript. All authors contributed to manuscript revision, read, and approved the submitted version.

## Conflict of Interest

The authors declare that the research was conducted in the absence of any commercial or financial relationships that could be construed as a potential conflict of interest.

## Publisher’s Note

All claims expressed in this article are solely those of the authors and do not necessarily represent those of their affiliated organizations, or those of the publisher, the editors and the reviewers. Any product that may be evaluated in this article, or claim that may be made by its manufacturer, is not guaranteed or endorsed by the publisher.

## Footnotes

1 This paper uses data from the National Educational Panel Study (NEPS): Starting Cohort Grade 5, doi: 10.5157/NEPS:SC3:8.0.1. From 2008 to 2013, NEPS data was collected as part of the Framework Program for the Promotion of Empirical Educational Research funded by the German Federal Ministry of Education and Research (BMBF). As of 2014, NEPS is carried out by the Leibniz Institute for Educational Trajectories (LIfBi) at the University of Bamberg in cooperation with a nationwide network.

## Appendix

**TABLE A:** Formulation of items – differentiation.

To what extent do the following statements apply to your German lessons in this class? Please tick a box in each line.
Does not apply at all [1], Does rather not apply [2], Partly [3], Does rather apply [4], Applies completely [5]
(d) I give students homework ranging in complexity based on their capability.
(e) I allow students who work faster to move on to the next assignment while I am still practicing or reviewing things with the ones that work slower.
(f) If students have difficulties in understanding, I give them additional assignments.
(g) I give more capable students extra assignments that are really challenging for them.

**TABLE B:** Formulation of items – grouping practices.

How often do you use the following social methods of learning in this German class? Please tick a box in each line.
Never [1], Once or twice per school year [2], Every few months [3], Every 2–4 weeks [4], Once per week [5], (Almost) every lesson
(a) Work with small student groups
(b) Partner work
(c) Discussion rounds
(e) Students acting as tutors (“Learning by Teaching,” peer tutoring)
(g) The class and I have discussions
(h) The students work on work sheets by themselves
(j) I explain something to the entire class

**TABLE C:** Pearson correlations of the predictor and outcome variables.

	1	2	3	4	5	6	7	8	9	10	11	12	13	14	15	16	17
(1) Differentiation	−																
(2) Small groups	0.15**	−															
(3) Partner work	0.06	0.32**	−														
(4) Discussion groups	0.14**	0.19**	0.15**	−													
(5) Peer tutoring	0.22**	0.18**	0.10**	0.18**	−												
(6) Joint discussion	0.08**	0.12**	0.10**	0.35**	0.09**	−											
(7) Individual work	0.05	-0.05	-0.04	0.04	-0.02	0.00	−										
(8) Frontal teaching	–0.01	0.01	0.06*	–0.01	0.05	0.01	0.12*	−									
(9) Class size	−0.43**	–0.02	0.08*	–0.04	–0.06	0.07*	–0.02	–0.03	−								
(10) Educational setting	0.43**	0.04	−0.06*	–0.01	0.02	−0.07*	–0.01	0.04	−0.81**	−							
(11) Percentage of female students within class	0.00	0.07	–0.01	0.02	0.01	–0.01	0.04	−0.07*	0.15**	−0.14**	−						
(12) Percentage of students with migration background within class	0.14**	0.04	0.06	–0.02	0.11**	–0.07	0.07	0.00	−0.14**	0.07	0.00	−					
(13) Percentage of students from academic households within class	−0.36**	0.07	0.03	–0.01	–0.07	0.10	–0.06	0.00	0.54**	−0.46**	0.13*	−0.33**	−				
(14) Teachers’ gender	0.09**	0.01	0.02	0.02	–0.01	–0.01	–0.02	–0.04	−0.11**	0.12**	0.01	0.02	−0.15*	−			
(15) Teachers’ migrant background	–0.07	–0.04	0.05	–0.01	0.01	0.04	–0.06	0.02	0.05	–0.09	–0.01	0.13*	0.01	0.08	−		
(16) Teachers’ years of experience	0.01	–0.02	0.06	–0.03	–0.06	0.00	–0.03	0.09	–0.03	0.02	0.03	–0.01	–0.12	0.04	–0.14	−	
(17) Teachers’ stated need for further training	–0.01	0.00	0.13**	0.05	0.03	0.09*	0.03	–0.04	0.11**	−0.15**	–0.01	0.04	0.00	0.09*	–0.08	0.07	−

*Intercorrelations based on the available data are presented below the diagonal. **p* < 0.05; ***p* < 0.01.*
